# Non-redundant association rules between diseases and medications: an automated method for knowledge base construction

**DOI:** 10.1186/s12911-015-0151-9

**Published:** 2015-04-15

**Authors:** François Séverac, Erik A Sauleau, Nicolas Meyer, Hassina Lefèvre, Gabriel Nisand, Nicolas Jay

**Affiliations:** 1 Laboratoire de Biostatistique et d’Informatique Médicale, Faculté de Médecine, Hôpitaux Universitaires de Strasbourg, Strasbourg, France; 2 Groupe Méthode en Recherche Clinique, Service de Santé Publique, Hôpitaux Universitaires de Strasbourg, Strasbourg, France; 3 LORIA – Equipe Orpailleurs, Université de Nancy, Nancy, France; 4 SPI-EAO, Faculté de Médecine, Université de Nancy, Nancy, France

**Keywords:** Data mining, Association rules mining, Natural language processing, Knowledge base

## Abstract

**Background:**

The widespread use of electronic health records (EHRs) has generated massive clinical data storage. Association rules mining is a feasible technique to convert this large amount of data into usable knowledge for clinical decision making, research or billing. We present a data driven method to create a knowledge base linking medications to pathological conditions through their therapeutic indications from elements within the EHRs.

**Methods:**

Association rules were created from the data of patients hospitalised between May 2012 and May 2013 in the department of Cardiology at the University Hospital of Strasbourg. Medications were extracted from the medication list, and the pathological conditions were extracted from the discharge summaries using a natural language processing tool. Association rules were generated along with different interestingness measures: chi square, lift, conviction, dependency, novelty and satisfaction. All medication-disease pairs were compared to the Summary of Product Characteristics, which is the gold standard. A score based on the other interestingness measures was created to filter the best rules, and the indices were calculated for the different interestingness measures.

**Results:**

After the evaluation against the gold standard, a list of accurate association rules was successfully retrieved. Dependency represents the best recall (0.76). Our score exhibited higher exactness (0.84) and precision (0.27) than all of the others interestingness measures. Further reductions in noise produced by this method must be performed to improve the classification precision.

**Conclusions:**

Association rules mining using the unstructured elements of the EHR is a feasible technique to identify clinically accurate associations between medications and pathological conditions.

## Background

In hospitals, an electronic health record (**EHR**) contains documents pertaining to one or several episodes of care for each patient. The EHR contains information organised in different sections, such as admission notes, medication lists, radiology reports, and complications. The widespread use of EHRs has led to the massive storage of clinical data. The amount of data being collected and stored is expanding rapidly. The term *big data* was coined to describe this evolving technology and science of data management. Data analysis is progressing that will enable organisations to convert this large amount of data into information and knowledge.

Accurate and complete knowledge of a patient’s pathological conditions and diagnoses is essential to optimise clinical decision-making. Knowledge of a patient’s pathological conditions is also critical for quality measurements, research and billing.

Following the seminal paper of Weed [1], the problem list aimed to occupy the central place in the medical reasoning process in a “problem-oriented medical record” (POMR). A problem list is a designated section of the patient’s medical chart that details all of the important medical information. In EHRs, a problem list can be used as a communication tool between physicians to understand a patient’s history. An accurate problem list is associated with a higher quality of care [2]. However, the concept of POMR is not completely efficient in today’s hospital information systems. Incomplete coding is a known limitation in hospital information systems, and problem lists are often inaccurate or incomplete [3,4].

Using drug-disease knowledge bases to infer pathological conditions from medications is a feasible and useful technique to complete the coding of pathological conditions. However, the manual construction of a database is a tedious task and is generally costly. Moreover, clinical knowledge is constantly evolving, practices change, and knowledge bases must be regularly updated. Data mining techniques, such as Association Rules Mining (**ARM**), have been successfully used to develop and update automated knowledge bases [5].

Another advantage of ARM is the possibility to describe the current practices at the level of a ward, a hospital or a higher level of aggregation (for example, an administrative area of care). For example, this method facilitates the identification of off-label uses.

Drug treatment information is generally well documented and easily accessible in organisations that have implemented computerised physician order entry. All medications are tracked for all patients, and the medications list is quite exhaustive.

Information pertaining to pathological conditions can be obtained from the unstructured elements of the EHR, such as “free text” clinical notes and discharge summaries. This difficult task can be achieved with natural language processing (**NLP**) tools [6,7]. Unstructured information recorded in the EHR contains rich information that could be useful to detect relevant clinical relationships between medications and pathological conditions. Many studies have shown the interests of using unstructured text of the EHR to identify adverse events [8-10].

Pathological conditions can also be obtained from the structured elements of the EHRs. Indeed, for billing purposes, every episode of care results in the coding of the pathological conditions treated during the stay. However, the discharge coding constrained by strict rules responds to an economic approach and does not always correspond with medical logic. Thus, gaps or non-relevant diagnoses can occur in the discharge coding list. Moreover, automated identification from EHRs using NLP is better for detecting postoperative complications compared with patient safety indicators based on discharge coding [11]. Structured information such as International Classification of Diseases, 10^th^ revision (ICD-10) codes seems to be inadequate to build patient cohort [9].

In this paper, we present a data driven method to develop a knowledge base relating drugs to pathological conditions through their therapeutic indications. Our approach relies on mining association rules in both the structured and unstructured data elements of the EHR. We describe our method in four steps in the following section. Then, some of our results are summarised. Finally, the discussion will follow with a brief general conclusion.

## Methods

Our study concerned all patients hospitalised between May 2012 and May 2013 in the department of Cardiology at the University Hospital of Strasbourg. Our method was divided into four main steps:

1. data-collection and pre-processing
2. association rules mining
3. post-processing and selection of association rules
4. method validation

Our dataset was split into a training set and a testing set. Seventy-five per cent of the dataset were randomly assigned to the training set and the 25% remaining to the testing set. The training set was used for the first three steps and the testing set was used for the method validation.

### Step 1: data collection and pre-processing

Two types of information were extracted from the EHR:

- Medication administered during the stay
- Medical conditions treated during the stay

First, the medications were extracted from EHRs. Each of the medications received by the patient was identified by its International Non-proprietary Name (INN) and was further linked to the Anatomical Therapeutic Chemical (ATC) classification system. Only light pre-processing was required. We excluded radio contrast agents (because we will not explore the radiological database) and electrolyte solutions (we considered that these medications will yield too much noise).

For medical problems, a NLP tool [12] (API FMTI from VIDAL©), which returns a list of ICD-10 codes, was used to parse the discharge summaries to extract pathological conditions.

**Figure 1:**
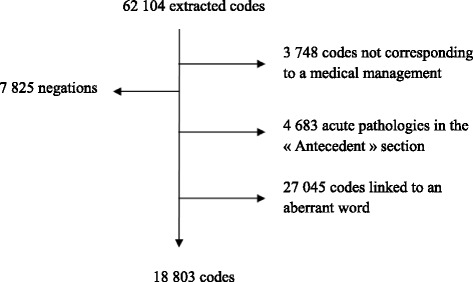
Cleaning diagnoses.

Data cleaning removed non-diagnosis and irrelevant ICD-10 codes. Four main procedures were executed (Figure 1).

1. All codes not corresponding to medical management were deleted. For example, all ICD-10 codes beginning with a “V” represent descriptive codes for traffic accidents and were deleted.
2. All codes retrieved from a negative sentence were also deleted.
3. All treatments administered during the stay are related to pathological conditions regarding the stay. Thus, all acute pathologies extracted in the section “Antecedents” were deleted.
4. Aberrant codes caused by homonymy due to several abbreviations in the discharge letter were also deleted.

Then, a database collection containing the ICD-10 codes corresponding to the pathological conditions and medications was created for each patient. In this database, the record lists all of the ICD-10 codes and all of the finest level codes in the ATC classification system for each medication a patient had received in the hospital. The data were obtained after approval from the CNIL, the French Data Protection Authority (authorisation number 1772715).

### Step 2: association rules building

Association rules aim to automatically extract interesting associations or correlations among a large set of items (attributes) that describes a set of objects in a database. In the database, sets of items can be characterised by its support (noted as *supp*), which is the proportion of objects sharing the attributes [13,14]. Association rules are in the form “L implies R”, which is noted as “L → R”. L (left, also called the antecedent) and R (right, also called consequent) are two sets of items that do not intersect. The interestingness of such rules can be characterised by many different measures to filter the “best” rules [13]. We used ARM to produce rules for correlations between a set of drugs and a set of medical conditions. The confidence (noted as *conf*) represents the conditional probability that an object includes items R, given that it includes items L. It is used to represent the reliability of the rule.

The rules were extracted with the Eclat-Z algorithm [14], which is a refinement of the well-known algorithm “Apriori” [15]. This algorithm computes a set of non-redundant and informative association rules in a very efficient way. However, the number of generated rules is generally very high and must be filtered according to user-defined criteria and usefulness measures. Because there is no clear evidence for choosing a unique measure, each of them has their own pros and cons. We focused on six measures [14,16].

1. The Chi square is computed from a 2 by 2 table. For the rule L → R, the counts in this table are the number of transactions containing L and R; L but not R (noted ┐ R); not L but R; and not R and not L. The formula is then:
  $$ \begin{array}{l}{\chi}^2\left(L\to R\right)=n{\left( lift\left(L\to R\right)-1\right)}^2 \times \\ {}\frac{conf\left(L\to R\right)\times supp\left(L\to R\right)\ }{\left( conf\left(L\to R\right)- supp\left(L\to R\right)\right)\times \left( lift\left(L\to R\right)- conf\left(L\to R\right)\right)}\end{array} $$
2. The lift (sometimes called interest) measures the simultaneous occurrences of the two sides of the rule.
  $$ lift\left(L\to R\right)=\frac{conf\left(L\to R\right)}{supp(R)} $$
3. The conviction measures the deviation of the rule L → R from the rule L → ┐ R.
  $$ conv\left(L\to R\right)=\frac{supp(L)\times supp\left(\neg R\right)}{supp(L)- supp\left(L\ \to\ R\ \right)} $$
4. The dependency measures the degree of independence between the events of each side of the rule (the fact that the occurrence of the antecedent is (or is not) dependent on the occurrence of the consequent).
  $$ dep\left(L\to R\right)=\left| conf\left(L\to R\right)- supp(R)\right| $$
5. The novelty is used to quantify the correlation between two attributes in a rule.
  $$ nov\left(L\to R\right)= supp\left(L\ \to\ R\right) - \left( supp(L)\times supp(R)\right) $$
6. The satisfaction is calculated with the conviction.
  $$ sat\left(L\to R\right)=\frac{conv\left(L\to R\right)-1}{conv\left(L\to R\right)} $$

### Step 3: post-processing of association rules

To achieve our objective, we selected association rules in which two conditions were fulfilled.

1. At least one medication was present in the antecedent.
2. At least one ICD-10 code was present in the consequent.

### Step 4: method validation

At the end of the three previous steps, we retrieved a set of non-redundant association rules satisfying several conditions that successfully passed several filters. The difficulty at this step was to assess the accuracy of those association rules in the absence of any existing reference. A solution was to extract the rules in which the antecedent represented only one medication and the consequent represented only one diagnostic. Using the therapeutic indications section of the Summary of Product Characteristics (SPC), the gold standard, we were able to label each of those rules as correct or incorrect. From this set of rules, the interestingness measures were calculated for each rule. Receiver Operating Characteristic (ROC) analyses were performed to identify the best cut-off for each interestingness measure. To assess the interest of each measure, recall (sensitivity), specificity, precision (positive predictive value), negative predictive value (NPV), exactness and area under curve (AUC) were calculated. Initially, we focused on six interestingness measures because there was no clear advantage to one measure. However, we also wanted to create a unique measure summarising the previous six measures. Using a logistic regression (the dependent variable was the correctness of the rule with respect to the SPC, which is the gold standard), a score based on the interestingness measures was constructed. Based on the ROC curve, the best cut-off score was determined. This method allowed us to build a new interestingness measure to select the best rules. All the previous process was performed on the training set. The cut-off of the interestingness measures were applied on the testing set. Only the cut-off of the Chi square was transformed. For taking into account the difference between the sample size of the training set and the testing set, we weighted the cut-off by the sample sizes ratio. We can see in the formula of the Chi square above that this interestingness measure depends on the size of the dataset. Finally, the indices (recall, specificity, precision,…) were extrapolated to the complete association rules database, including multi-element rules (with several items in the antecedent or in the consequent).

### Software

Medication extraction was performed with Business Object, the software used for medication requests at the University Hospitals of Strasbourg. All our processes were performed using the Coron Data Mining Platform (http://coron.loria.fr/site/index.php) and the Language and Environment for Statistical Computing: R (R Core Team, R Foundation for Statistical Computing, Vienna, Austria. 2012, available at http://www.R-project.org/).

## Results

### Population

The population database consisted of 1,440 discharge letters and the corresponding medications. The training set contains 1080 stays (*n*_*train*_) and the testing set the remaining 360 (*n*_*train*_). From the letters of the training set, 62,104 ICD-10 code diagnoses were extracted. After cleaning, 18,803 codes remained with only 1,063 distinct codes (up to the 5^th^ digit). For medications, 11,162 items (each item corresponds to a line in the medical order) were extracted with 363 different medications.

### Association rules

In total, on the training set, 214,309 association rules were retrieved by the Eclat-Z algorithm, among which 99,312 fulfilled the filter of at least one medication in the antecedent and at least one ICD-10 code in the consequent. Among this last set, 910 rules fulfilled the type {one medication} → {one ICD-10 code}.

### Method validation

With respect to the SPC gold-standard, on the 910 rules, 53 were valid (see Table 1: the top ten rules).

**Table 1:** **Top 10 medication-problem associations under score**

Medication	Problem	Score	GS evaluation
Digoxin	Atrial fibrillation	6.11	True
Levothyroxine Sodium	Hypothyroidism	4.58	True
Human Insulin	Diabetes	4.51	True
Insulin Glargine	Diabetes	4.48	True
Phytomenadione	Atrial fibrillation	4.44	False
Fluindione	Atrial fibrillation	4.31	True
Dabigatran Etexilate	Atrial fibrillation	4.04	True
Nicardipine	Hypertension	3.05	True
Amiodarone	Atrial fibrillation	3.03	True
Furosemide	Atrial fibrillation	2.18	False

The quality measures are summarised in Table 2. The ranges of the different measures were variable. For example, lift and conviction ranged from approximately 0 to 10 for an expected range of 0 to ∞, and chi square ranged from 0 to approximately 500. In contrast, the ranges for novelty and satisfaction were very narrow compared to their expected ranges (respectively −1 to 1 and –∞ to 1). We sorted the available rules according to each quality measure.

**Table 2:** **Summary of the quality measures on the association rules, {one medication} → {one ICD-10 code} database**

	Lift	Conviction	Chi square	Novelty	Dependency	Satisfaction	Score
Min	0.602	0.635	0	−0.064	0	−0.574	−07.287
25%	0.970	0.995	0.502	−0.002	0.010	−0.005	−4.320
50%	1.104	1.018	2.217	0.004	0.023	0.018	−3.8144
75%	1.311	1.059	8.826	0.01	0.052	0.056	−3.0574
Max	8.601	11.883	521.294	0.116	0.689	0.916	7.576
Mean	1.202	1.096	8.826	0.005	0.047	0.042	−3.491
SD	0.464	0.516	27.021	0.012	0.073	0.139	1.289

SD: Standard deviation.

**Figure 2:**
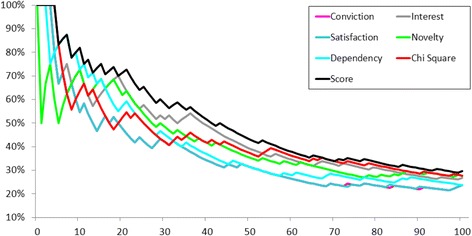
Progressing exactness for quality measures on the 100 first association rules, {one medication} → {one ICD-10 code} database.

To select the truly accurate rules, we calculated the proportion of exact rules among the n best rules for each measure. Figure 2 presents these proportions for up to n = 100. If 40 rules were selected, the exactness will be approximately 60% according to all quality measures (except interest, which is much lower). Globally, above 20 rules, the exactness was approximately 10% for all quality measures (except for interest).

The score, created with a logistic regression, ranged from −7.3 to 7.6 with a median of −3.8 (Table 2). Figure 2 demonstrates that all the quality measures and the score had similar values for selecting less than 15 rules. Dependency, satisfaction and the score performed better than other measures for selecting between 15 and 25 rules. The score was better for selecting more than 25 rules.

ROC curves for each interest measure were performed on the testing set to determine a cut-off. These thresholds were applied on the testing set and allowed the calculation of several indices (Table 3). The threshold of the Chi square was transformed as follow: $Threshol{d}_{test}=\frac{n_{test}}{n_{train}} \times Threshol{d}_{train}$.

**Table 3:** **Evaluation of interestingness measures**

IM	Recall	Specificity	Precision	NPV	Exactness
Chi^2^	0.69	0.82	0.20	0.98	0.80
Lift	0.74	0.72	0.15	0.98	0.72
Conv	0.74	0.69	0.14	0.98	0.69
Dep	0.76	0.69	0.14	0.98	0.70
Sat	0.74	0.69	0.14	0.98	0.69
Nov	0.71	0.78	0.18	0.98	0.78
Score	0.74	0.83	0.27	0.98	0.84

IM: interestingness measures; NPV: negative predictive value.

The dependency presented the best recall (0.76), but the composite score yielded the best results for all other indices. The score yielded a precision of 0.27, while the other measures ranged from 0.16 to 0.20. The score yielded an exactness of 0.84, while the other measures ranged from 0.69 to 0.80.

## Discussion

We propose a method for building accurate association rules from an EHR by linking different pathological conditions and medications. After data preparation, association rules were built and then post-processed to retrieve the relevant and operational rules. We validated our method on a subset of association rules (one medication implies one disease code). Our primary goal is to create a complete set of rules, containing multi-elements rules (including several items in the antecedent or in the consequent). The final goal is to provide a tool to improve drug-disease knowledge databases. The results we present here tested the application of this method on 1,440 patients. Indeed, we developed a tool based on quality measures to rank the rules. However, these ranked rules must be reviewed by a human (physician) for definite validation, in the absence of any existing reference for multi-elements rules. Although the accuracy of the multi-elements rules is difficult to assess, these rules are promising. For example, if we select the rules containing hypertension in the consequent, we find in the top ranked rules associations between hypertension and furosemide or amlodipine alone, but we find also multi-elements rules. For example: Myocardial infarction, Bisoprolol → Hypertension. This rule could express the fact that Bisoprolol is the preferential treatment in hypertensive patients who have had a myocardial infarction. Because a final human step is required, one can object that this method could be replaced by rules built only by human experts. However, this process would take a long time and be extremely costly. Moreover, we assume that a human team working with a ranked list of association rules is much more efficient than building rules from scratch. Our method yields several improvements. First, our method yields a set of rules already created. Second, these rules are ranked on an accuracy measure.

Finally, on the subset of association rules used for the validation of the method, we retrieved 53 rules describing clinical relationship between medications and their indications. This doesn’t seem much but we worked with a small sample of only 1,440 stays. This allows us to perform an error analysis of the method validation. The understanding of misclassification is critical to improve the method. Different types of error lead to the misclassification of a rule:

- Relevant rules describing a relationship different from indication. Three rules were describing an adverse event of a medication (For example: Bisoprolol → nausea and vomiting).
- Irrelevant rules indicating a transitive association (For example: Metformin → Hypertension). When there is a strong comorbidity between two conditions, for example hypertension and diabetes, we can have a consequent part of the patients presenting the two conditions and the two treatments. This can lead to transitive association.
- Irrelevant rules resulting from the noise produced by the extraction of pathological conditions.

Compared with similar published methods, this method is applied to a very different type of dataset. For example, Wright and al. [17] worked on a database consisting of 100,000 patients with 272,749 health conditions and 442,658 medications. In comparison, our complete database was approximately 100 times smaller (1,440 patients). However, our database was only 10 times smaller for conditions (25,180 ICD codes) and 30 times smaller for medications. When considering only the different codes, the 1,756 conditions for Wright and al. becomes 1,170, and 2,128 medications becomes 396. Because we worked in a specific hospital ward (which was specialised), we retrieved proportionally less different medications than in a whole hospital and also retrieved fewer diagnoses. The fact that there were the same number of codes between a hospital data set (100,000 patients) and a cardiologic database (1,400 patients) indicates that our data may be noisier. Indeed, we worked with text mined diagnoses from discharge letters and not with structured information gathered in EHR. These considerations could explain why the quality measures were not as good as expected. Our future goal is to reduce the noise generated by this method to improve the classification precision. It will be interesting to compare this method with the association rules built with diagnoses extracted from structured elements of the EHR. These methods could be compatible.

To prune the abundance of association rules, we searched and deleted redundant rules according the inclusion of an item in another rule. An alternative method (or an additional feature) could be to generalise the association rules [18] with a one pass algorithm for the creation of rules (for example, see [19]) or by filtering already created rules [20]. The requirement for using such methods is to have a hierarchy of items (a taxonomy), and the primary underlying idea is that if a relevant rule concerns a given item, the similar rules involving its children in the hierarchy are not relevant. In our case, we disposed of two different taxonomies:

1. For ICD-10 codes, each five-digit code belongs to a category. Each category belongs to a group of categories, which belongs to a chapter.
2. For medications, the ATC classification is a hierarchy of five levels.

Currently, generalised association rules require further research to improve our method.

To validate our method, we used only disease codes and medications from one specialised hospital ward to have a more homogeneous medical practice. Using this validated method, the next step is to build rules based on the whole hospital. Moreover, all the structured items in the EHR, such as laboratory results or data imaging are candidates for association rules mining.

## Conclusions

Data mining in the unstructured elements of EHRs (as free text) is a feasible technique for the identification of accurate associations between medications and pathological conditions. The creation of a composite score based on several interestingness measures demonstrated promise for the selection of the best rules. Furthermore, the relevant rules could be applied in various ways to improve quality of care.

## Abbreviations

EHR: Electronic health record
ARM: Association rules mining
NLP: Natural language processing
INN: International non-proprietary name
ATC: Anatomical therapeutic chemical
ICD-10: International classification of diseases 10^th^ revision
SPC: Summary of product characteristics
ROC: Receiver operating characteristic
NPV: Negative predictive value
AUC: Area under curve
